# Evaluating virtual reality for anxiety reduction in children and adolescents with life-limiting conditions: a randomized pilot study

**DOI:** 10.1186/s12904-026-02152-7

**Published:** 2026-05-20

**Authors:** Anna Šebová, Iveta Fajnerová, Lenka Drnková, Lucie Hrdličková

**Affiliations:** 1 Department of Psychiatry, 1st Faculty of Medicine, Charles University and General University Hospital, Prague, Czech Republic; 2 Center for Virtual Reality Research in Mental Health and Neuroscience, National Institute of Mental Health, Klecany, Czech Republic; 3 Department of Psychiatry and Medical Psychology, 3rd Faculty of Medicine, Charles University, Prague, Czech Republic; 4 Department of Pediatrics, Motol and Homolka University Hospital, 2nd Faculty of Medicine, Charles University, Prague, Czech Republic; 5 Pediatric Supportive Care Team, Motol and Homolka University Hospital, Prague, Czech Republic; 6 Center for Palliative Medicine, 2nd Faculty of Medicine, Charles University, Prague, Czech Republic

**Keywords:** VR, Virtual reality, Pediatric palliative care, Symptom management, Anxiety

## Abstract

**Background:**

Children and adolescents with life-limiting conditions (LLCs) often face distressing medical procedures, prolonged hospital stays, and other challenges, leading to heightened anxiety and psychological distress. To address these symptoms, psychological interventions using modern technologies like immersive virtual reality are recommended alongside traditional treatments. While virtual reality (VR) has shown promise in reducing distress in various pediatric groups, its use in pediatric palliative care (PPC) is still emerging. The aim of this study was to assess the feasibility, acceptability and effectiveness of experiential VR intervention and compare it with a second distraction method in the form of a video in a population of pediatric patients with LLC.

**Methods:**

This pilot randomized control trial used a crossover design with a pre- and post- intervention assessment of anxiety, fear, and pain using standardized scales. Post-session patient-reported data were also collected using supplementary items. A total of eighteen pediatric patients (8 girls, 11–17 years of age;10 boys, 10 − 17 years of age) participated in the study, randomized into two groups. The experimental group (*N* = 10) received VR in both sessions, while the control group (*N* = 8) experienced a passive video in the first session, followed by VR in the second. Given the characteristics of the sample, non-parametric statistical analyses were conducted using the Mann-Whitney U test and Wilcoxon signed rank test.

**Results:**

The findings showed that VR distraction produced a statistically significant reduction in anxiety on all three occasions it was administered across both groups (experimental group: *p* = 0.028, *r* = 0.773; *p* = 0.049, *r* = 1.000; control group: *p* = 0.028, *r* = 0.905), demonstrating a consistent effect across the sample. Video distraction showed a trend toward anxiety reduction that did not reach statistical significance (*p* = 0.091), while producing a significant reduction in pain (*p* = 0.027). Among participants who received both interventions, VR was rated as significantly more enjoyable than video distraction (*p* = 0.036, *r* = -1.00). Most participants wanted to be distracted during the procedure (91%), felt that it decreased their anxiety (73%), found the VR system easy to use (96%), and wanted to use it again in future care (88%).

**Conclusions:**

These exploratory findings from a pilot RCT provide preliminary evidence that VR distraction may serve as a feasible, acceptable, and potentially efficacious complementary approach for managing anxiety in children and adolescents with life-limiting conditions. While video distraction produced broadly comparable outcomes across measures, a differential pattern was observed for the predefined primary outcome of anxiety, which warrants confirmation in a future adequately powered trial. These findings highlight the potential of distraction-based interventions to provide meaningful therapeutic benefit in this population. The significantly higher enjoyment ratings for VR and its strong acceptability further support its potential clinical value in pediatric palliative care, where positive experience and engagement are central to quality of life. Nevertheless, the practical burden of implementation and associated costs require further investigation before definitive recommendations can be made.

**Trial Registration:**

The study was registered retrospectively 8.1. 2025 (ISRCTN50308167).

## Background

Children and adolescents with life-limiting diagnoses often deal with frequent hospital stays, numerous unpleasant medical procedures, treatment side effects, and other challenges. This can lead to higher levels of anxiety, prevalence of persistent “sadness,” and other mental health issues among these children [1, 2].

To address this, it is recommended to complement traditional medical approaches, such as pharmacological anxiety relief, with psychological interventions aimed at alleviating psychological symptoms and enhancing the patient’s quality of life [2, 3]. One innovative form of intervention is the use of modern technologies such as immersive virtual reality (VR). Immersive VR is a computer-generated environment that creates a realistic illusion that makes users feel as if they are truly in the virtual world [4]. Thus, patients can divert their attention from pain, anxiety, or fear associated with long-term hospital stay or unpleasant medical procedures [5–7].

VR has been shown to be effective in various pediatric settings. For example, hemato-oncological diseases [6, 8], burn treatment [9], routine medical procedures such as blood draws [7], associated needle procedures [5, 10], and cannulation [11, 12]. VR-distraction therapy (VR-DT) has even been shown to be superior to standard hospital distraction practices such as toys, television, books, and parental comfort [8]. Another advantage is that VR not only affects the child patient but can also be used to reduce anxiety in the parent or caregiver [13].

The available literature includes articles primarily from adult care [14, 15], but also specifically focused on PPC [16]. Currently, there is only one publication that directly focuses on the use of VR in PPC. This case study highlights the potential of VR with a vision to create a specialized intervention program utilizing VR within the therapeutic support that palliative care can offer families and their children [16]. Johnson et al. [17], in a feasibility study, highlighted the vast potential of VR as an innovative treatment method, providing quality-of-life improvements for palliative care patients while also decreasing their reliance on pharmacological treatments. VR has the ability to address a range of symptoms in palliative care patients [14, 18]; however, more adequately powered randomized controlled trials (RCTs) in this area are still needed.

Based on the lack of published studies in this area, we conducted our study with the primary aim of assessing the feasibility, acceptability, and efficacy of VR for easing anxiety in children with LLC. The second objective was to compare the efficacy of VR with a simple video-viewing experience used as a control condition.

## Methods

### Study design

**Fig. 1:**
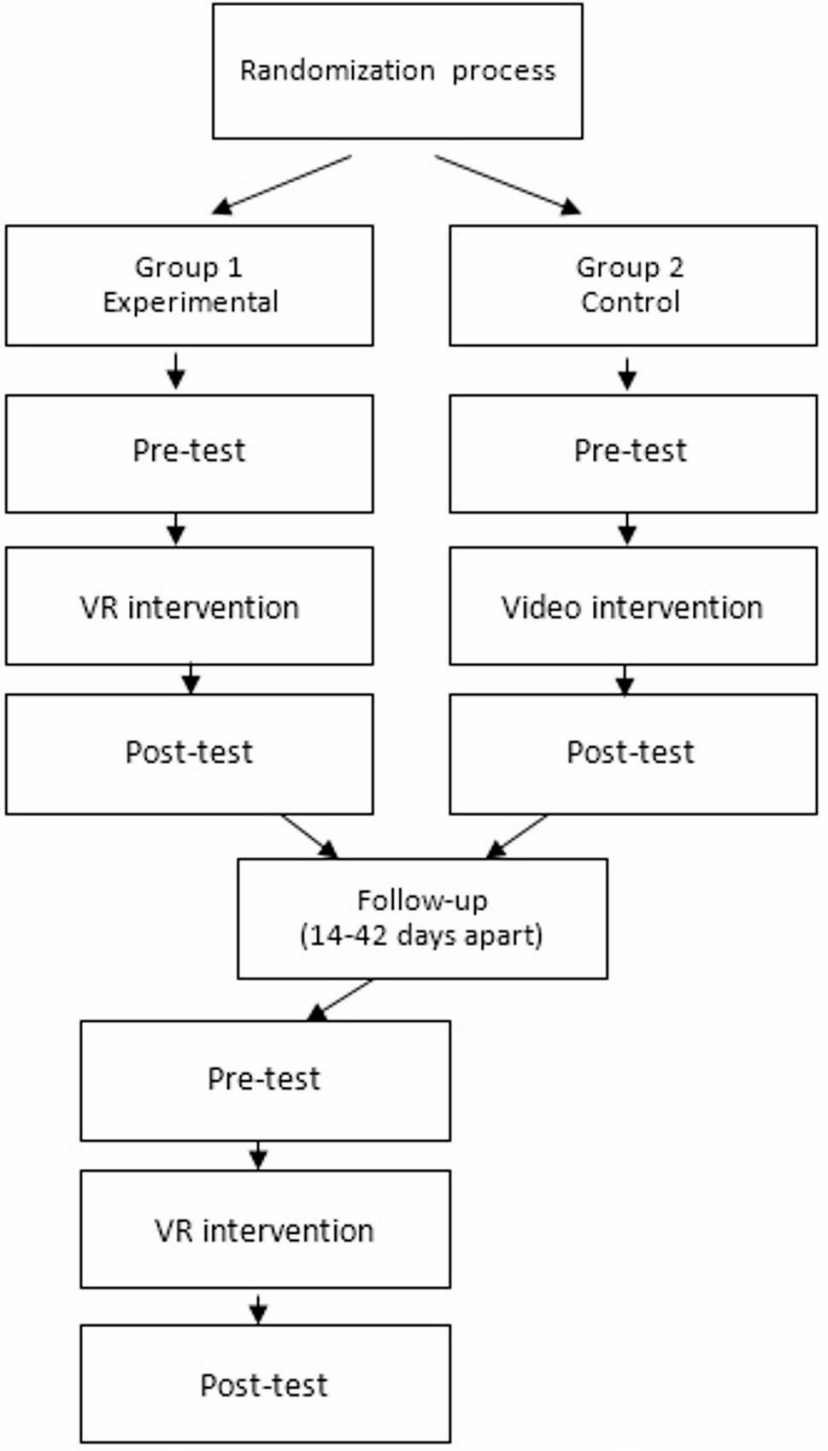
An overview of the design of the study

This pilot randomized control trial study employed both between-subjects and within-subjects crossover design to evaluate both the feasibility of virtual reality distraction intervention (VR-DT) and its efficacy compared to a video-based distraction method. Participants, children, and adolescents (*N* = 18) were recruited by opportunity sampling and subsequently randomly assigned to either an experimental group (*N* = 10), which received two VR intervention sessions spaced 14–42 days apart, or a control group (*N* = 8), which first experienced a video intervention followed by a VR intervention in the second session. The random assignment of participants to the experimental (VR) and control (video) groups was carried out using a simple draw method. Participant names were written on individual slips of paper and randomly drawn from an envelope without replacement. Pre- and post-intervention measurements were taken for the observed constructs, including anxiety, pain, and fear. Figure 1 provides an overview of the study’s design.

### Setting

The study was conducted at the Motol and Homolka University Hospital in close cooperation with the Pediatric Supportive Care Team, which provides palliative care to pediatric patients and their families in all hospital departments. Pediatric patients from hematology-oncology and gastroenterology outpatient clinics participated in this study. VR distraction intervention was administered during chemotherapy sessions in the hemato-oncological unit and during infusion treatments in the gastroenterological unit. A researcher was present during the procedures to ensure protocol adherence, oversee the intervention, and monitor the patient’s condition. Data collection began with the first participant in June 2023, and ended with the last participant in December 2023.

### Study population

**Fig. 2:**
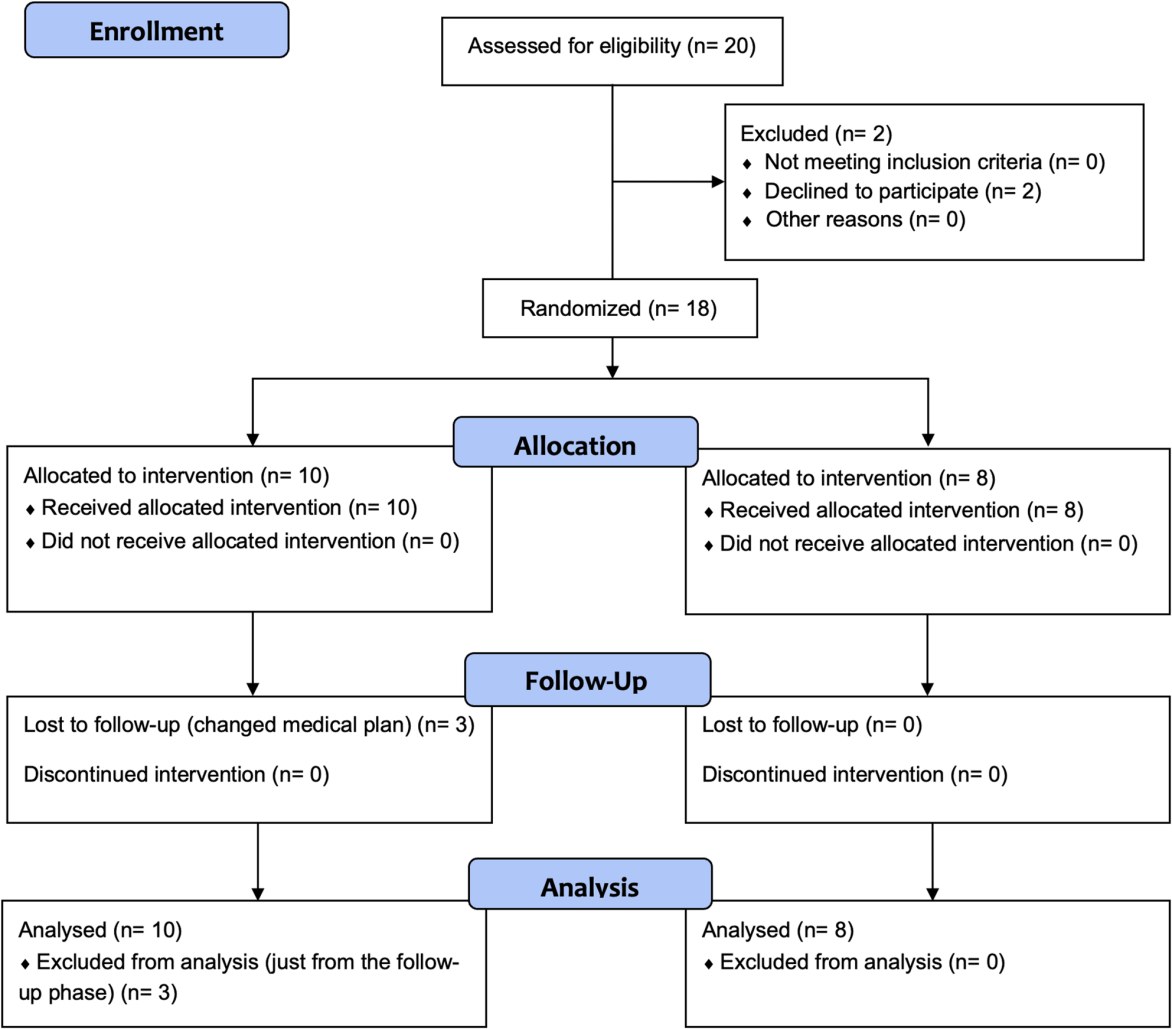
Consort diagram

The study population consisted of pediatric and adolescent patients aged 10–17 with hemato-oncological or gastroenterological conditions. These conditions were selected because they are life-limiting and require extensive, recurring medical treatment. This makes affected children suitable candidates for intervention. Inclusion criteria, contained (a) sufficient level of contact with the environment and ability to cooperate, (b) fluency in Czech for both children and parents, (c) diagnosis of a life-threatening or life-limiting illness according to the internationally accepted list of palliative relevant diagnoses [19], and (d) being a 10–17,9 years old, (e) haven’t a vision or mental problem at a level to watch the video, (f) voluntarily agreeing to participate in the study. The exclusion criteria were (a) age below 10 or above 18 years, (b) unstable health status, (c) inability to speak Czech, and (d) absence of parental consent for participation in the study. Eighteen patients were initially enrolled in the study, with fifteen completing both sessions and three patients were unable to participate in the second session due to changes in their medical plan. See Fig. 2 for the CONSORT diagram detailing participant enrollment, allocation, follow-up, and analysis. The sample size was guided by practical considerations, such as the size of the accessible participant pool, clinical setting limitations, and the exploratory nature of the pilot RCT study. The parents of all included children and adolescents signed a consent form for their participation in the study, which was approved by the Ethics Committee of the Motol and Homolka University Hospital (EK − 667/22) in accordance with the Declaration of Helsinki (2013). Data collection began with the first participant in June 2023, and ended with the last participant in December 2023. See Fig. 2 for details.

### Assessment and intervention procedures

The assessment and intervention procedures involved several steps. Each VR/video session was standardized to a total duration of 35 min, comprising 10 to 15 min of VR or video exposure administered concurrently with chemotherapy or infusion, 8 min allocated for pre-session questionnaire completion, and 6 min for post-session assessments.

During both the PRE and POST sessions, the following scales were used: the Children’s Fear Scale (CFS), measuring the current level of fear; the Wong-Baker Faces Pain Rating Scale (WBFPRS), used to evaluate perceived pain; and the situational anxiety subscale of an established anxiety inventory, which captured fluctuations in anxiety levels over time. The Children’s Medical Fear Scale (CMFS) was administered only during the PRE session as part of a repeated-measures framework to establish baseline levels of medical-related fear. The Simulator Sickness Questionnaire (SSQ) was administered exclusively during the POST session, and only after the VR intervention, to assess symptoms of cybersickness following VR exposure. Moreover, additional non-standardized questions were used. Prior to VR sessions, participants were asked regarding their prior experience with video games and VR technology. Specific inquiries were made regarding previous encounters with VR in a hospital setting. Following both VR and video sessions, participants provided feedback on their level of engagement, enjoyment, and sense of presence in the experience. Additionally, respondents expressed their level of agreement with statements regarding their desire for distraction during medical procedures and the perceived efficacy of VR or video in alleviating anxiety after the procedure. (For more information see subsection Outcome Measures below)

Throughout the VR experience, the researcher provided assistance with the VR equipment (Meta Quest 2 headset), addressed any queries from the participants, and, in case of emergencies, helped to manage stress and discomfort of the participants. The same procedure but only with video was performed in the first session, in the control group using an Apple iPad. The topics of the videos matched two of the VR apps; thus, participants could choose from an underwater world or the flora and fauna of Antarctica. Both the videos were landscape shots with background music.

Three VR environments were utilized: (1) *Ocean Rift*, which features an underwater world and marine life (a game widely employed in existing research, such as [6, 11], (2) *National Geographic* scenes (e.g., kayaking in Antarctica), and (3) *ISS Tour VR*, offering a virtual tour of the International Space Station. Furthermore, owing to the presence of an intravenous (IV) line in the patients’ arms from the gastroenterological unit, making the use of the National Geographic app impractical, an alternative VR application was introduced to accommodate these specific circumstances. The alternative third app was *Notes on Blindness*, an app that enables users to experience life without vision. This adaptation ensured that patients in this unit could still benefit from immersive VR experiences, despite the constraints imposed by their medical treatment.

### Outcome measures

The primary outcome was predefined as change in anxiety levels from pre- to post-intervention, measured using “Scale measuring anxiety in children – short version”. Secondary outcomes included change in fear and pain levels measured by standardized visual scales. Other pre-specified outcomes included level of cybersickness, usability of the VR, and the subjective feedback of the participants including the level of sense of presence, enjoyment and engagement. See details below.

As a pilot RCT, no formal sample size power analysis was conducted, as the primary aim was to assess feasibility and generate preliminary effect size estimates to inform the design of a future full-scale trial.

#### Children Fear Scale -CFS [20]

The Children’s Fear Scale (CFS), developed by McMurtry et al. in 2011, was used to assess self-reported fear. The scale consists of five faces illustrating escalating fear intensities, rated from 0 (no fear) to 4 (extreme fear). A Czech version was prepared by the research team through a double-translation (forward and back-translation) process; however, formal validation of this translation is pending.

#### Wong-Baker Faces Pain Rating Scale – WBFPRS [21]

Pain intensity was measured using the Wong-Baker FACES Pain Rating Scale (WBFPRS), developed by Wong and Baker in 1983. The WBFPRS consists of six facial expressions ranging from 0 (no pain) to 10 (worst pain) and is widely validated in pediatric populations. An official Czech translation of the WBFPRS used in this study is available through the Wong-Baker FACES Foundation; however, formal psychometric validation of the Czech version has not been published.

#### Child Medical Fear Scale – CMFS[22]

Fear of medical procedures was assessed using the Child Medical Fear Scale (CMFS), originally developed by Broome in 1991 and subsequently updated by Broome and Mobley in 2003. The CMFS is a 17-item self-report instrument designed to measure fear related to medical experiences in children. Each item is rated on a 3-point Likert scale (0 = not afraid, 1 = a little afraid, 2 = very afraid), with total scores ranging from 0 to 34. Higher scores reflect higher levels of medical fear. For this study, the CMFS was translated into Czech by the research team using a double-translation and reconciliation method; however, formal psychometric validation of the Czech version has not yet been completed.

####  Scale Measuring Anxiety in Children - Short Version [23]

The original version of the scale was developed by Slovak authors Müllner et al. in 1983 and revised for the Czech context by Toman in 2018. It is a self-report screening inventory measuring anxiety in children aged 10 to 15, using two subscales: state anxiety and trait anxiety. Each subscale contains 17 items, and responses are rated on a 4-point scale (1 – not true, 4 – true). Scores on this subscale range from 15 (minimum) to 60 (maximum).

####  Simulation Sickness Questionnaire – SSQ [24]

The Simulator Sickness Questionnaire (SSQ) was developed by Kennedy, Lane, Berbaum, and Lilienthal in 1993 to quantify symptoms of simulation sickness. The SSQ comprises 16 symptoms rated on a 4-point Likert scale (0 = none, 1 = slight, 2 = moderate, 3 = severe). Factor analysis of the SSQ has identified three primary symptom clusters: Nausea, Oculomotor, and Disorientation. Each cluster is weighted differently to compute subscale scores and an overall total score, which reflects the severity of simulation sickness experienced. The SSQ has been widely adopted in research involving virtual reality (VR) systems to assess cybersickness. The Czech translation of the SSQ was conducted by the Center for Virtual Reality Research in Mental Health and Neuroscience at the National Institute of Mental Health in the Czech Republic, and an official validation paper is currently in preparation.

####  System usability scale – SUS [25]

The System Usability Scale (SUS), developed by Brooke in 1996, is a standardized and widely validated tool for assessing the usability and acceptability of technological systems. For the purposes of this study, two items were selected from the full scale: “I would like to use this system repeatedly” (P1) and “The system is easy to use” (P2), both rated on a 1–5 agreement scale (1 = strongly agree to 5 = strongly disagree). These items were administered following each VR session only, as they specifically concern the usability of the VR system rather than the video intervention.

#### Additional Questions

In addition to standardized measures, participants were asked to respond to several custom-designed questions developed by the research team. These items explored prior experience with video games and VR technology, as well as participants’ subjective feedback on their level of engagement, enjoyment, and sense of presence during the intervention. Responses to these items were recorded using a structured 4-point scale (0 = not at all to 3 = significantly). Furthermore, participants rated their agreement with statements regarding their desire for distraction during medical procedures and the perceived effectiveness of VR or video in reducing anxiety, using a 5-point Likert scale (1 = strongly agree to 5 = strongly disagree). As no suitable existing questionnaire was available to measure these specific aspects in children, the items were created by the research team and tested in pilot study in 2022. This pilot feasibility study confirmed that the items were age-appropriate and easily understood by the target population.

### Data analysis

Statistical analyses were conducted using JASP version 0.16.1. Given the characteristics and limited size of the sample, non-parametric statistical methods were employed, as they do not require the assumption of normal data distribution. The Wilcoxon signed-rank test was used for within-subject comparisons—specifically, to assess changes in anxiety, fear, and pain levels before and after the intervention in both the experimental and control groups (analyzed separately across the two sessions). The Mann–Whitney U test was applied for between-group comparisons in two cases: [1] to compare outcome measures between the experimental (VR) and control (video) groups in Session 1, and [2] to compare perceived enjoyment between the two sessions between the groups (VR and video). These statistical methods were selected to match the nature of the data and ensure valid, reliable results. All tests were one-tailed, with a significance level set at *α* = 0.05.

## Results

The VR procedure was well accepted by all involved children and adolescents and can be evaluated as feasible with regard to its implementation. None of the participating volunteers interrupted the VR session and there was no refusal to participate before the second session due to VR procedure. Cybersickness, a commonly reported concern with VR use, was minimal to absent across the sample, further supporting the safety and tolerability of the intervention in this population.

The results are divided into three sections: the first subsection describes the demographic data of the sample, the second part focuses on the comparison between the pre- and post-intervention phases within each group, and in the third part, we compare the two groups.

### Demographic data

The mean age of the sample was 14.39 years (*SD* = 2.20), and in total eight girls and ten boys participated in the study. Of the 18 participants, eight were being treated for hemato-oncological conditions and ten for gastroenterological conditions. (For more information see Table 1.)

**Table 1:** Demographic data of the sample

Variable	VR Group	Video Group	Total
Gender			
Female	5	3	8
Male	5	5	10
Age			
Mean (SD)	14.5 (2.37)	14.25 (2.12)	14.39 (2.20)
Median	14.5	14.5	14.5
Minimum	10	11	10
Maximum	17	17	17
Unit			
Hemato-oncology	4	4	8
Gastroenterology	6	4	10

*VR* Virtual reality, *SD * Standard deviation

### Within subject comparison

The observed level of anxiety in the experimental group was significantly lower after the VR intervention (1st session: *Mdn* = 17.00, 2nd session: *Mdn* = 15.00) than at the initial assessment (1st session: *Mdn* = 19.50, 2nd session: *Mdn* = 16.50) in both sessions (*p* < 0.05). However, the reduction in fear and pain was not statistically significant. See Table 2. for details.

**Table 2:** Wilcoxon signed-rank test comparing the amount of anxiety, fear and pain before and after the intervention procedure in the experimental group measured in both sessions

**Measure**	**Session**	**Pre M (SD)**	**Post M (SD)**	**W**	**z**	**p**	**r**
Anxiety	VR1	26.9 (14.12)	24.2 (14.22)	39.00	1.955	0.028	0.773
	VR2	17.9 (15.32)	16.6 (14.29)	10.00	1.826	0.049	1.000
Fear	VR1	0.40 (0.52)	0.10 (0.32)	6.00	1.604	0.074	1.000
	VR2	0.43 (0.53)	0.29 (0.49)	4.00	0.535	0.386	0.333
Pain	VR1	0.70 (0.82)	0.50 (0.71)	10.00	0.674	0.286	0.333
	VR2	0.86 (0.90)	0.57 (0.79)	3.00	1.342	0.173	1.000

The table shows the three components of distress – anxiety (measured by Scale Measuring Anxiety in Children - Short Version), fear (measured by Children Fear Scale), and pain (measured by Wong-Baker Faces Pain Rating Scale)

*M* Mean, *SD* Standard deviation, *W* Wilcoxon signed-rank test statistic, *z* Standardized test statistic, *p* Probability value, *r* Effect size, *VR1* Virtual Reality Session 1, *VR2* Virtual Reality Session 2

In the control group, only the amount of pain was significantly lower after video intervention (*Mdn* = 0.00) compared to the initial measurements (*Mdn* = 1.50), *p* < 0.05; for details, see Table 3. No significant reductions were observed in the other two variables in the first session (Table 3). However, in the second session, when the control group underwent VR intervention, the level of anxiety was significantly lower after VR intervention (*Mdn* = 17.00) compared to the initial assessment (*Mdn* = 19.50), *p* < 0.05. The same was observed for pain, where the level before the VR intervention was higher (*Mdn* = 2.00) than after (*Mdn* = 0.50), *p* < 0.05. The reduction in fear was not significant in any of the sessions. (Table 3).

**Table 3:** Wilcoxon signed-rank test comparing the amount of anxiety, fear and pain before and after the intervention procedure in the control group in both sessions

Measure	Session	Pre M (SD)	Post M (SD)	W	z	*p*	*r*
Anxiety	Video (S1)	23.0 (6.76)	20.8 (6.92)	6.00	1.604	0.091	1.00
	VR	22.8 (9.15)	20.4 (7.90)	20.00	1.992	0.028	0.905
Fear	Video (S1)	0.38 (0.52)	0.13 (0.35)	3.00	1.342	0.173	1.00
	VR (S2)	0.75 (1.39)	0.13 (0.35)	3.00	1.342	0.186	1.00
Pain	Video (S1)	1.25 (0.89)	0.38 (0.52)	15.00	2.023	0.027	1.00
	VR (S2)	2.38 (2.77)	0.88 (0.99)	10.00	1.826	0.049	1.00

The table shows the three components of distress – anxiety (measured by Scale Measuring Anxiety in Children - Short Version), fear (measured by Children Fear Scale), and pain (measured by Wong-Baker Faces Pain Rating Scale)

*M *Mean, *SD* Standard deviation, *W* Wilcoxon signed-rank test statistic, *z* Standardized test statistic, *p* Probability value, *r* Effect size, *Video (S1)* Video Session 1, *VR (S2)* Virtual Reality Session 2

### Between subject comparison

No statistically significant differences were found between the experimental and control groups for the measured variables (Table 4).

**Table 4:** Mann-Whitney U test comparing observed measures in experimental and control group in session 1

Measure	Group	Pre M (SD)	Post M (SD	W (pre)	*p* (pre)	W (post)	*p* (post)
Anxiety	VR	26.9 (14.1)	24.2 (14.22)	42.00	0.447	41.50	0.464
	Video	23.0 (6.76)	20.75 (6.92)				
Fear	VR	0.40 (0.52)	0.10 (0.32)	41.00	0.479	39.00	0.597
	Video	0.38 (0.52)	0.13 (0.35)				
Pain	VR	0.70 (0.82)	0.50 (0.71)	26.00	0.915	42.50	0.418
	Video	1.25 (0.89)	0.38 (0.52)				

The table shows the three components of distress – anxiety (measured by Scale Measuring Anxiety in Children - Short Version), fear (measured by Children Fear Scale), and pain (measured by Wong-Baker Faces Pain Rating Scale)

*M* Mean, *SD* Standard deviation, *W* Wilcoxon signed-rank test statistic, *p* Probability value, *VR* Virtual Reality group, *Video* Video group

The additional measures (non-standardized questions) revealed that the VR (*Mdn* = 3.00) intervention was rated as more enjoyable than the video distraction (*Mdn* = 2.00) by patients who experienced both interventions (*p* < 0.05, for details, see Table 5). No significant differences in enjoyment were observed between the first session with VR and the second session with VR.

**Table 5:** Wilcoxon signed-rank test for the comparison of enjoyment perceived in the two sessions in the VR and Video groups

**Measure**	**Session**	**M (SD)**	**W**	**z**	**p**	**r**
Enjoyment	Video (S1)	2.38 (0.52)	0.00	-1.826	0.036	-1.00
	VR (S2)	2.88 (0.35)				
	VR 1	2.50 (0.71)	4.00	0.535	0.773	
	VR 2	2.43 (0.79)				

A structured 4-point scale was used to measure the level of enjoyment

*M* Mean, *SD* Standard deviation, *W* Wilcoxon signed-rank test statistic, *z* Standardized test statistic, *p* Probability value, *r* Effect size, *Video (S1)* Video Session 1, *VR (S2)* Virtual Reality Session 2, *VR 1* Virtual Reality Session 1, *VR 2* Virtual Reality Session 2

The subjective feedback obtained from the children, incorporating items from the validated System Usability Scale [25] alongside supplementary feasibility indicators, highlights the good acceptance and positive perception of the VR system during medical procedures. The majority of participants in the study: rated the VR system as easy to use (96%), would like to use VR repeatedly (88%), wanted to be distracted during medical procedure (91%), and believed that the distraction helped alleviate their anxiety during the procedure (73%).

No significant differences were observed between the male and female subjects or based on the underlying diagnosis (hematological-oncological unit vs. gastroenterological unit). The only notable difference was related to the appropriateness of specific VR applications based on procedural requirements. For instance, the VR application *National Geographic - Travel to Antarctica* was deemed unsuitable for pediatric gastroenterology patients, as it necessitates greater mobility, which is incompatible with the immobility required during infusion procedures.

## Discussion

The present pilot RCT study provides preliminary evidence for the feasibility, acceptability, and potential efficacy of VR distraction intervention in alleviating anxiety in children and adolescents with life-limiting conditions. Most children and adolescents found VR distraction beneficial. They appreciated having a distraction during the medical procedure, felt that it helped reduce their anxiety, expressed a interest in using the system again, and found it easy to use overall.

Our findings align with existing evidence supporting VR as an effective method for reducing anxiety in pediatric populations [4, 6, 10]. However, the results indicate that VR-DT is not conclusively superior to video distraction in terms of efficacy, which is consistent with the findings of Tennant et al. [8]. In our study, both immersive VR and video interventions produced reductions across distress measures, though a differential pattern emerged for the predefined primary outcome of anxiety — VR consistently achieving statistical significance while video distraction showed a trend toward reduction that did not reach significance. Both modalities likely operate through overlapping mechanisms: redirecting attention away from discomfort, engaging users through stimulating audiovisual content, and imposing a level of cognitive load that occupies mental resources, potentially reducing the brain’s capacity to process pain and anxiety signals [26, 27]. However, VR’s higher level of immersion and interactivity may amplify these effects beyond what passive video can achieve — a distinction that warrants further investigation to better understand the specific added benefits of immersive technologies in distress management.

A related question concerns whether comparable efficacy between the two interventions reflects genuine equivalence or whether it was shaped by the design of the control condition itself. The video was intentionally matched in content to the VR experience — for example, pairing an immersive ocean world VR environment with a passive ocean-themed video accompanied by music — in order to isolate the effect of interactivity and immersion rather than content novelty. This methodologically sound approach may nonetheless have narrowed the performance gap between conditions more than would be observed in routine clinical practice, where video distraction is typically far less curated. Crucially, among participants who had experienced both interventions, VR was rated as significantly more enjoyable than video distraction (*p* = 0.036, *r* = -1.00). This finding carries clinical relevance in pediatric palliative care, where positive experience and engagement are central to quality of life. This is further supported by the strong feasibility and acceptability data, with the majority of participants rating the system as easy to use (96%) and expressing a wish to use it again in future care (88%).

These findings are further supported by existing literature. Tennant et al. [8] similarly reported that VR outperformed iPad distraction across all subjective measures, while Oh et al. [13] found that most children wanted distraction, were willing to use VR again, found it easy to use, and considered it effective in reducing anxiety.

A notable gap addressed by this study is the limited research on VR interventions, specifically within PPC. To date, only one case study by Weingarten et al. [16] examined VR usage in this context. Although the research is more extensive in adult palliative care (e.g [14, 15, 17], it remains relatively underrepresented compared to other fields, such as oncology (e.g [6, 8]. Existing evidence suggests significant potential for VR in palliative care [17]. Beyond its utility as a tool for distraction, VR offers the capacity to deliver immersive and emotionally resonant experiences that promote comfort, serenity, and moments of joy for individuals approaching the end of life [14]. These interventions can mitigate distress and enhance well-being, thereby complementing conventional approaches to symptom management [17].

This study contributes to the body of knowledge by exploring the basis for effective implementation of VR interventions in this area and the target population. Implementing VR.

interventions in PPC involves a range of practical and logistical considerations that are essential for successful and sustainable integration. While most commercial VR systems are user-friendly and require minimal technical expertise, staff still need basic training to operate the equipment, troubleshoot common issues, and support children—especially those unfamiliar with or apprehensive about the technology. This includes understanding safety protocols, such as monitoring for cybersickness, ensuring hygienic maintenance of headsets, and overseeing safe use in confined spaces.

Practical barriers include the physical size and weight of headsets for smaller children, the need for initial calibration, difficulties accommodating medical equipment such as IV lines or oxygen masks, and the time required for disinfection and maintenance between uses. From a cost perspective, although the initial investment in VR hardware and software may appear substantial, the potential benefits — including decreased reliance on sedatives [28] and improved patient experience [29] — suggest promising long-term cost-effectiveness. The increasing availability of low-cost standalone VR systems — such as Meta Quest or Pico — and freely accessible immersive content further supports feasibility even in resource-limited settings. That said, the practical burden of maintaining a VR setup must be properly weighed against simpler alternatives such as video distraction or access to streaming platforms.

A relevant practical consideration concerns the sustainability of VR’s benefits over time. Notably, in the experimental group, VR produced a statistically significant reduction in anxiety across both sessions, with the effect size increasing rather than diminishing from the first to the second session (*r* = 0.773 to *r* = 1.000), providing preliminary support that the observed benefits were not simply attributable to the novelty of the technology. While two sessions are insufficient to draw definitive conclusions about long-term sustainability, this pattern suggests that the therapeutic effect may be maintained — or even strengthened — across repeated use, possibly as children become more comfortable and confident with the technology. That said, whether these effects persist across many more sessions over a longer care trajectory remains an open and important question that future research should specifically address.

Current literature mainly compares VR with standard care or non-digital distractions (e.g., toys and verbal interaction) [6, 30]. Inspired by Tennant et al. [8], our study introduces video-based distraction as a control condition, enabling a direct comparison of two digital interventions with similar content — for example, an ocean world theme — but differing in sensory engagement, contrasting a flat screen with a fully immersive experience. This represents a novel methodological contribution to the field.

### Limitations

This pilot RCT should be interpreted in light of several limitations inherent to its design and scope. The small sample size (*N* = 18) was an intentional feature of the pilot design, though it does restrict statistical power and the extent to which findings can be generalized. Even statistically significant results should be understood as preliminary, as effect sizes derived from small samples may shift in larger cohorts. The unequal group sizes (experimental *n* = 10, control *n* = 8) are also worth acknowledging in this context. Taken together, these characteristics reflect the nature of pilot work — the primary aim was signal detection and feasibility assessment, and so the findings are best understood as a foundation for a future full-scale RCT.

Several aspects of the study design are also worth acknowledging. As is common in VR intervention research, full blinding of participants and researchers to condition allocation was not possible, which may have introduced an element of performance or observer bias. It is also possible that part of the anxiety reduction observed in the VR group reflects the novelty of the technology itself, particularly for participants with limited prior VR experience, rather than the therapeutic mechanism of immersive distraction alone. Whether this effect would persist across repeated sessions is a question the current pilot was not designed to address, and it represents a meaningful direction for future investigation.

Regarding the crossover design, control participants experienced passive video distraction before transitioning to VR in the second session. Their responses to VR may therefore have been shaped in part by prior familiarity with the procedural setting and the earlier intervention, which is worth bearing in mind when interpreting the results of that arm of the study.

The design of the control condition is also worth acknowledging. The video was intentionally content-matched to the VR experience in order to control for novelty and isolate the effect of immersion and interactivity. While methodologically sound, this approach may have narrowed the performance gap between conditions more than would be observed in routine clinical practice, where standard video distraction is typically far less curated. This should be considered when interpreting the comparable efficacy findings between the two interventions.

The use of self-report measures to assess anxiety, fear, and pain, while standard practice and appropriate for this population, carries the possibility of social desirability bias. Complementing self-report with objective physiological indicators, such as heart rate variability or cortisol levels, in future studies would provide additional depth and corroboration to the findings.

The composition of the sample also introduces considerations around generalizability. Participants presented with a range of life-limiting conditions, which reflects the real-world complexity of pediatric palliative care as a clinical field, where no single diagnosis predominates and care is provided across a wide spectrum of conditions. While restricting recruitment to a single diagnosis would have increased sample homogeneity, it would also significantly limit the clinical relevance and generalizability of findings to real-world pediatric palliative care practice. Nonetheless, it is possible that the experience and effectiveness of VR distraction varies across diagnostic groups in ways the current sample size was not sufficient to explore, and future research should examine whether VR distraction is differentially effective across specific diagnostic categories within this population.

Finally, the inclusion of children aged 10 and above was based on findings from a prior feasibility pilot study conducted by our research team, in which younger children experienced difficulties navigating the VR system independently. While this was a considered and empirically informed decision, it means that findings cannot be extended to younger children, who may equally benefit from non-pharmacological distraction during procedures. Exploring VR solutions suitable for this younger age group, including age-appropriate content and simplified navigation, constitutes an important direction for future research.

### Future research

Several directions for future research emerge from this work. Most immediately, a future full-scale RCT with adequate statistical power and recruitment across multiple centers is needed to confirm these preliminary findings. Such a trial would benefit from a more homogeneous sample — for example, focusing on a specific diagnostic subgroup within pediatric palliative care — or alternatively from sufficient sample size to enable meaningful subgroup analyses across diagnostic categories, thereby addressing the inherent heterogeneity of this clinical population. Future studies should also aim to include younger children, as they represent a substantial portion of the pediatric population with life-limiting conditions — an important gap given that the current study was restricted to children aged 10 and above. Exploring VR solutions specifically designed and evaluated for younger age groups therefore remains a priority.

Future research would benefit from incorporating qualitative methods — such as semi-structured interviews or focus groups with children, adolescents, and their families — to provide deeper insight into the lived experience of VR distraction in a pediatric palliative care context. Such approaches would complement quantitative outcome data by capturing the nuanced emotional, relational, and experiential dimensions of VR use that numerical scales alone cannot fully convey. Qualitative exploration would also offer a valuable opportunity to invite participants to reflect on and compare their experiences of VR and video distraction in their own words, providing deeper understanding of what distinguishes the two modalities from the patient perspective — an dimension that the current study was unable to fully capture through numerical ratings alone. Together, these insights would inform the development of more person-centred and contextually sensitive distraction interventions in pediatric palliative care.

Further research is also warranted to explore the efficacy of specific VR intervention types — such as VR relaxation applications — in contrast with distraction-based approaches, as these may operate through different mechanisms and offer distinct therapeutic benefits. Comparing VR with other immersive technologies such as augmented reality could offer additional insights into the relative effectiveness of different approaches. The development and testing of customizable or interactive VR content tailored to individual preferences and clinical needs could further improve patient engagement and therapeutic outcomes.

Formal cost-effectiveness analyses and implementation models that address both technical and clinical barriers would help broaden VR’s reach across diverse pediatric care environments. Future studies should also examine the long-term impact of VR on anxiety and emotional well-being over time, providing insight into whether therapeutic effects are sustained as the novelty of the technology diminishes. Finally, incorporating physiological outcome measures — including heart rate variability and cortisol levels — alongside deeper qualitative exploration of the VR experience in a palliative care context would considerably enrich understanding of both the mechanisms and the lived experience of these interventions.

## Conclusion

This pilot RCT provides preliminary evidence that VR distraction was associated with a significant reduction in anxiety in children and adolescents with life-limiting conditions, contributing to a field where research remains scarce. Video distraction showed a non-significant trend toward reduced anxiety, with the limited sample size precluding definitive comparisons. Although it was associated with a reduction in pain, this finding should also be interpreted cautiously given the small sample. Taken together, the findings suggest that distraction-based interventions — whether immersive or passive — carry meaningful therapeutic value in this population.

Both interventions were feasible and well tolerated in this population, and among children who experienced both, VR was rated as more enjoyable than video distraction, highlighting its potential to enhance engagement in PPC where patient experience is central. These findings support the feasibility and potential clinical value of VR distraction in pediatric palliative care and provide a foundation for the design of a future adequately powered RCT.

## Data Availability

The study data are not publicly available, but can be provided upon reasonable request.

## Abbreviations

VR: Virtual reality
VR: DT: Virtual Reality Distraction Therapy
PPC: Pediatric palliative care
LLC: Life-limiting condition
